# Can germ cell neoplasia in situ be diagnosed by measuring serum levels of microRNA371a-3p?

**DOI:** 10.1007/s00432-017-2490-7

**Published:** 2017-08-17

**Authors:** A. Radtke, J.-F. Cremers, S. Kliesch, S. Riek, K. Junker, S. A. Mohamed, P. Anheuser, G. Belge, K.-P. Dieckmann

**Affiliations:** 1 Faculty of Biology and Chemistry, University of Bremen, Leobener Str. 2, 28359 Bremen, Germany; 2 Department of Clinical and Surgical Andrology, Centre of Reproductive Medicine and Andrology, University of Muenster, Muenster, Germany; 3 Department of Pathology, Klinikum Bremen-Mitte, Bremen, Germany; 4 Department of Cardiac and Thoracic Vascular Surgery, University of Luebeck, Luebeck, Germany; 5 Department of Urology, Albertinen Krankenhaus, Hamburg, Germany

**Keywords:** Germ cell tumour, microRNA, Germ cell neoplasia in situ, Testicular biopsy, Quantitative polymerase chain reaction

## Abstract

**Purpose:**

Diagnosing germ cell neoplasia in situ (GCNis) can detect germ cell tumours (GCTs) at the pre-invasive stage. To date, testicular biopsy with the potential of surgical complications is the only way of safely diagnosing GCNis. Recently, microRNAs (miRs) 371-3, and miR 367 were shown to be valuable serum biomarkers of GCTs. We explored the usefulness of these candidate miRs as a marker for GCNis.

**Methods:**

27 patients with GCNis and no concomitant GCT were enrolled. All patients underwent measuring serum levels of miR-371a-3p and miR-367-3p before treatment, 11 had repeat measurement after treatment, 2 also had testicular vein blood examinations. Serum levels were measured by quantitative PCR. In addition, four orchiectomy specimens of patients with GCT were examined immunohistochemically and by in situ hybridization (ISH) with a probe specific for miR-371a-3p to look for the presence of this miR in GCNis cells.

**Results:**

The median serum level of miR-371a-3p was significantly higher in patients with GCNis than in controls, miR-367 levels were not elevated. Overall, 14 patients (51.9%) had elevated serum levels of miR-371a-3p. The highest levels were found in patients with bilateral GCNis. Levels in testicular vein serum were elevated in both of the cases. After treatment, all elevated levels dropped to normal. In two orchiectomy specimens, miR-371a-3p was detected by ISH in GCNis cells.

**Conclusions:**

Measuring miR-371a-3p serum levels can replace control biopsies after treatment of GCNis. In addition, the test can guide clinical decision making regarding the need of testicular biopsy in cases suspicious of GCNis.

## Introduction

Germ cell neoplasia in situ (GCNis; formerly called carcinoma in situ testis, CIS, or testicular intraepithelial neoplasia, TIN) is the uniform precursor of all adult testicular germ cell tumours (GCTs) (Rajpert-De Meyts et al. 2016). GCNis is thought to develop from embryonic germ cells and is thus present in the testis many years before the clinical manifestation of the GCT (Dieckmann and Skakkebaek 1999). Diagnosing GCNis represents a method of early detection of GCT at the pre-invasive stage. Practically, the only way of diagnosing the lesion is testicular biopsy with immunohistochemical examination of the specimen (Hoei-Hansen et al. 2007a, b). The diagnostic accuracy of a two-site biopsy is high with a more than 90% specificity and a somewhat lower sensitivity of about 80–85% (Dieckmann and Skakkebaek 1999; Dieckmann et al. 2007). Clinically, testicular biopsies for diagnosing GCNis are considered valuable in populations with high risk of GCT (Rajpert-De Meyts et al. 2016), mainly patients with unilateral GCT (search for contralateral tumour) and infertile men. Although testicular biopsy is involved with a low burden of complications of less than 3%, it represents an invasive surgical procedure with the potential of adverse events (Dieckmann et al. 2011). Therefore, alternative methods for the detection of GCNis have been investigated ever since the introduction of biopsies into clinical practice. However, the diagnostic accuracy of semen-based examinations (Hoei-Hansen et al. 2007a, b; van Casteren et al. 2008a, b; Almstrup et al. 2011) and imaging procedures (Lenz et al. 1996; Holm et al. 2001; Tsili et al. 2013), proved to be unfavourably low, so far.

Recently, the microRNA (miR) clusters miR371-3 and miR302/367 have been suggested as valuable new serum tumour markers of GCT (Murray and Coleman 2012; Belge et al. 2012). Two major studies revealed a very high sensitivity of 86% and a specificity of 92% of miR-371a-3p (van Agthoven and Looijenga 2016; Dieckmann et al. 2017). Contrary to the classical GCT markers, beta-human chorionic gonadotropin (b-HCG), alpha-fetoprotein (AFP) and lactate dehydrogenase (LDH), miR-371a-3p is expressed both in seminoma and nonseminoma (Murray et al. 2016). As miR-371a-3p is highly specific for GCT and as the precursor lesion is morphologically quite similar to seminoma cells (Donner et al. 2004), it could be hypothesized that GCNis does also express the new marker. If so, measuring these miRs in serum would constitute a non-invasive avenue for searching for GCNis.

To explore the utility of measuring serum levels of the candidate microRNAs for diagnosing GCNis, we first looked to serum levels of candidate microRNAs in patients with GCNis at several points of time. We then tried to morphologically identify miRs in GCNis cells by employing simultaneously in situ hybridization (ISH) and immunohistochemical techniques. We restricted our analysis to miR-371a-3p and miR-367-3p because these two microRNAs had previously been shown to be the most sensitive candidate miRs for detection of GCT (van Agthoven and Looijenga 2016).

## Materials and methods

### Patients for serum investigations

As GCNis may present clinically with at least four different features (Berney et al. 2016), we made the following stratifications regarding the patient population:

(Group 1) GCNis in a solitary testis, no contralateral testis is present at the time of examination, (Group 2) GCNis in one testis and a healthy testis is present on the contralateral side, (Group 3) both testes afflicted with GCNis, and (Group 4) one GCNis-afflicted testis is accompanied by a GCT-bearing contralateral one. As any GCT tissue (primary tumour or metastasis) represents a significant source of miR production, we recruited patients exclusively with GCNis only for the present study. Thus, patients with any concurrent GCT tissue at the time of examination (group 4, vide supra) were excluded from this study to avoid confounding of serum levels by invasive malignancy.

Cubital vein serum was obtained from 27 patients aged 35.3 ± 8.8 years (Table 1). Seventeen patients were recruited prospectively from Albertinen-Krankenhaus Hamburg during 2012–2016, 15 of whom had contralateral biopsy in the presence of unilateral GCT, and 2 had bilateral biopsies for evaluation of testicular microlithiasis. Ten patients were enrolled from a serum biobank of the University of Muenster, Department of Andrology, all of the latter had been examined for male infertility during 2005-2016. Six of the patients had been briefly reported earlier (Spiekermann et al. 2015). According to the stratification listed above, groups 1-3 consisted of 11, 14, and 2 patients. All of the 11 patients of group 1 had undergone previous orchiectomy for a contralateral GCT. The interval between orchiectomy for GCT and acquisition of the serum sample ranged from 2 to 31 days (Table 1). According to published experience regarding the decay of miR levels after orchiectomy, clearance of tumour-induced miR expression was expected to be completed after these intervals (Spiekermann et al. 2015). All of the 11 group 1 patients had clinical stage 1 disease, i.e. no metastases were detected and all had normal classical tumour markers. Eleven patients (four of group 1, five of group 2 and two of group 3) had repeat measurements of serum levels of microRNAs after treatment of GCNis. Treatment comprised local radiotherapy to the testis with 13–20 Gy in three cases, chemotherapy with carboplatin in one case, and orchiectomy of the afflicted testis in seven cases. Testicular vein serum was additionally obtained from two patients of group 2 during surgery.

**Table 1:** Study population (patients)

No.	Age (yr)	Group	Contralateral testis
1	52	1	Orchiectomy 3 d before testing: seminoma CSI
2	47	1	Orchiectomy 3 d before testing: seminoma CSI
3	25	1	Orchiectomy 31 d before testing: seminoma CSI
4	45	1	Orchiectomy 2 d before testing: seminoma CSI
5	52	1	Orchiectomy 30 d before testing: seminoma CSI
6	44	1	Orchiectomy 4 d before testing: seminoma CSI
7	45	1	Orchiectomy 3 d before testing: seminoma CSI
8	33	1	Orchiectomy 3 d before testing: seminoma CSI
9	26	1	Orchiectomy 25 d before testing: seminoma CSI
10	26	1	Orchiectomy 29 d before testing: seminoma CSI
11	33	1	Orchiectomy 9 d before testing: seminoma CSI
12	30	2	Not tumour-bearing
13	31	2	Not tumour-bearing
14	26	2	Not tumour-bearing
15	16	2	Not tumour-bearing
16	34	2	Not tumour-bearing
17	34	2	Not tumour-bearing
18	32	2	Not tumour-bearing
19	36	2	Not tumour-bearing
20	39	2	Not tumour-bearing
21	35	2	Not tumour-bearing
22	33	2	Not tumour-bearing
23	47	2	Not tumour-bearing
24	27	2	Not tumour-bearing
25	33	2	Not tumour-bearing
26	40	3	GCNis bilateral
27	33	3	GCNis bilateral

*CSI* clinical stadium I, *d* days, *GCNis* germ cell neoplasia in situ, *yr* years

Serum samples of 20 healthy men or individuals with non-malignant testicular disease aged 37.5 ± 10.8 years served as controls, all of whom had been reported previously (Dieckmann et al. 2017).

In all cases, except for the two testicular vein samples, serum samples were derived from cubital vein blood aspiration. Serum was obtained after centrifugation and it was then stored deep frozen at −80 °C until processing.

### Patients for histological investigation of presence of microRNAs in GCNis cells

Four archival orchiectomy specimens of patients with testicular GCTs were analysed by immunohistochemistry and in situ hybridisation (ISH) to look for the presence of miR-371a-3p in GCNis cells neighbouring the invasive tumours. The patients were aged 28.0 ± 0.7 years. Histologically, the GCTs comprised mixed nonseminomatous tumours in two cases, one pure seminoma, and one pure embryonal carcinoma, respectively.

All patients enrolled for the various parts of this study had given informed consent to the examination. Ethical approval of the study was provided by Aerztekammer Bremen (reference No 301, 2011).

### Measurement of serum levels of miRNA

For isolation of miRNA, 200 µl of serum was processed with the miRNeasy mini kit (Qiagen, Hilden, Germany) according to the manufacturer’s instructions. Then 6 µl of RNA was reverse transcribed using the TaqMan MicroRNA Reverse Transcription Kit (Applied Biosystems, Darmstadt, Germany) and preamplified using Real-Time Ready cDNA Master (Roche Diagnostics, Mannheim, Germany). TaqMan assays (Applied Biosystems, Darmstadt, Germany) for miR-371a-3p (assay ID 002124), miR-367-3p (assay ID 000555) and the endogenous control miR-93-5p (assay ID 000432) were used in a 1:100 dilution for the preamplification. The qPCR was performed on a 7500 Fast Real-Time PCR System (Applied Biosystems, Damstadt, Germany) using FAST Start Universal Probe Master (Roche Diagnostics, Mannheim, Germany) and the undiluted TaqMan Assays. Finally, the relative quantity (RQ) was calculated according to the 2^−∆∆CT^ method (Livak and Schmittgen 2001). As cut-off value for miR-371a-3p serum levels, a RQ = 5 was used to differentiate between GCNis and controls corresponding to the method employed previously (Dieckmann et al. 2017).

### Immunohistochemistry

For morphological identification of GCNis, four orchiectomy specimens with invasive GCTs were randomly selected from the pathology archive. Of the FFPE-blocks, sections of 5 µm were obtained from tumour-free parts of the specimen. Staining with haematoxylin and eosin with standard histological techniques were used to confirm tumour-free areas and to identify tubules containing GCNis cells. Oct4 staining (Diagnostic BioSystems, Pleasanton, CA, USA) was then conducted according to institutional standard operating procedures (Jones et al. 2004; Berney et al. 2015) to safely ascertain GCNis cells.

### miRNA in situ hybridization

Sections with clear immunohistochemical evidence of GCNis were then processed with in situ hybridisation with a miRCURY LNA probe (Exiqon, Vedbaek, Denmark; probe ID 38555-15) specific for miR-371a-3p. The protocol was performed according to the manufacturer’s instructions using a proteinase K concentration of 15 µg/ml, a hybridisation temperature of 51 °C and a probe concentration of 80 nM. Microscopic evaluations were performed on an Axioskop 2 plus microscope (Zeiss, Göttingen, Germany) at 100× to 200× magnifications. Histological findings were documented using the AxioCam HRc digital camera (Zeiss, Göttingen, Germany) and then edited with AxioVision Software v.4.8 (Zeiss, Göttingen, Germany). Presence of miR-371a-3p within GCNis cells was defined by distinct blue staining of intratubular cells, and accordingly, only these cells were considered miR-371a-3p positive. Only the presence or absence of the miR-371a-3p in the specimen was evaluated, no quantification was attempted.

### Statistical methods

The Mann–Whitney *U* test was used to compare median miRNA expressions among the various subgroups. The Wilcoxon signed-rank test was used for comparison of dependent subgroups. Categorical data was analysed with the Fisher’s exact test. Significance was assumed at *p* < 0.05. Exact 95% confidence intervals of proportions were calculated.

## Results

### Serum level measurements

Table 2 shows the individual results of measurements of miR-371a-3p and miR-367-3p of all of the 27 patients. Serum levels of miR-371a-3p were elevated in 14 of 27 patients (51.9%; exact 95% confidence interval 31.9–71.3%). The relative frequencies of miR-371a-3p expression above the cut-off value of the various GCNis subgroups and controls are presented in Table 3. All subgroups were significantly different from controls (Table 3) but differences among the groups were not significant, statistically (all *p* > 0.1).

**Table 2:** Individual results of serum levels of miR-371a-3p and miR-367-3p before and after treatment

No.	Group	RQ in cubital vein before treatment		RQ in cubital vein after treatment		RQ in testicular vein before treatment	
		MiR-371a-3p	MiR-367-3p	MiR-371a-3p	MiR-367-3p	MiR-371a-3p	MiR-367-3p
1	1	0.00	0.00	na	na	na	na
2	1	0.00	0.00	na	na	na	na
3	1	25.69	0.00	0.00	3.36	na	na
4	1	5.22	21.37	0.00	0.62	na	na
5	1	0.00	1.61	0.00	0.00	na	na
6	1	52.38	0.87	na	na	na	na
7	1	34.83	0.00	0.00	0.00	na	na
8	1	18.24	0.00	na	na	na	na
9	1	37.27	0.00	na	na	na	na
10	1	0.00	0.31	na	na	na	na
11	1	38.52	0.00	na	na	na	na
12	2	0.00	0.00	0.00	0.00	na	na
13	2	0.00	58.37	0.00	0.00	na	na
14	2	22.11	325.17	na	na	na	na
15	2	8.28	2.11	na	na	na	na
16	2	3.76	0.00	na	na	na	na
17	2	0.00	6.20	na	na	na	na
18	2	93.60	0.00	0.00	0.00	na	na
19	2	0.35	0.00	5.98	0.00	na	na
20	2	0.00	0.00	0.00	63.67	57.26	11.49
21	2	0.00	0.00	na	na	na	na
22	2	0.00	0.00	na	na	na	na
23	2	1.61	0.00	na	na	295.42	0.00
24	2	40.10	0.00	na	na	na	na
25	2	23.57	0.00	na	na	na	na
26	3	35.80	0.00	9.06	0.00	na	na
27	3	237.18	0.00	24.08	0.00	na	na

*na* not available, *RQ* relative quantity

**Table 3:** Relative proportions of miR-371a-3p expression in different subgroups

Subgroup	Positive (*n*)	Total (*n*)	Positive (%)	95% CI (%)	Comparison with controls (*p* value)
All GCNis	14	27	51.9	31.9–71.3	0.001
Group 1	7	11	63.6	30.8–89.1	0.0009
Groups 2 and 3	7	16	43.8	19.8–70.1	0.012
Controls	1	20	5.0	0.1–24.9	–

*CI* confidence interval

**Fig. 1:**
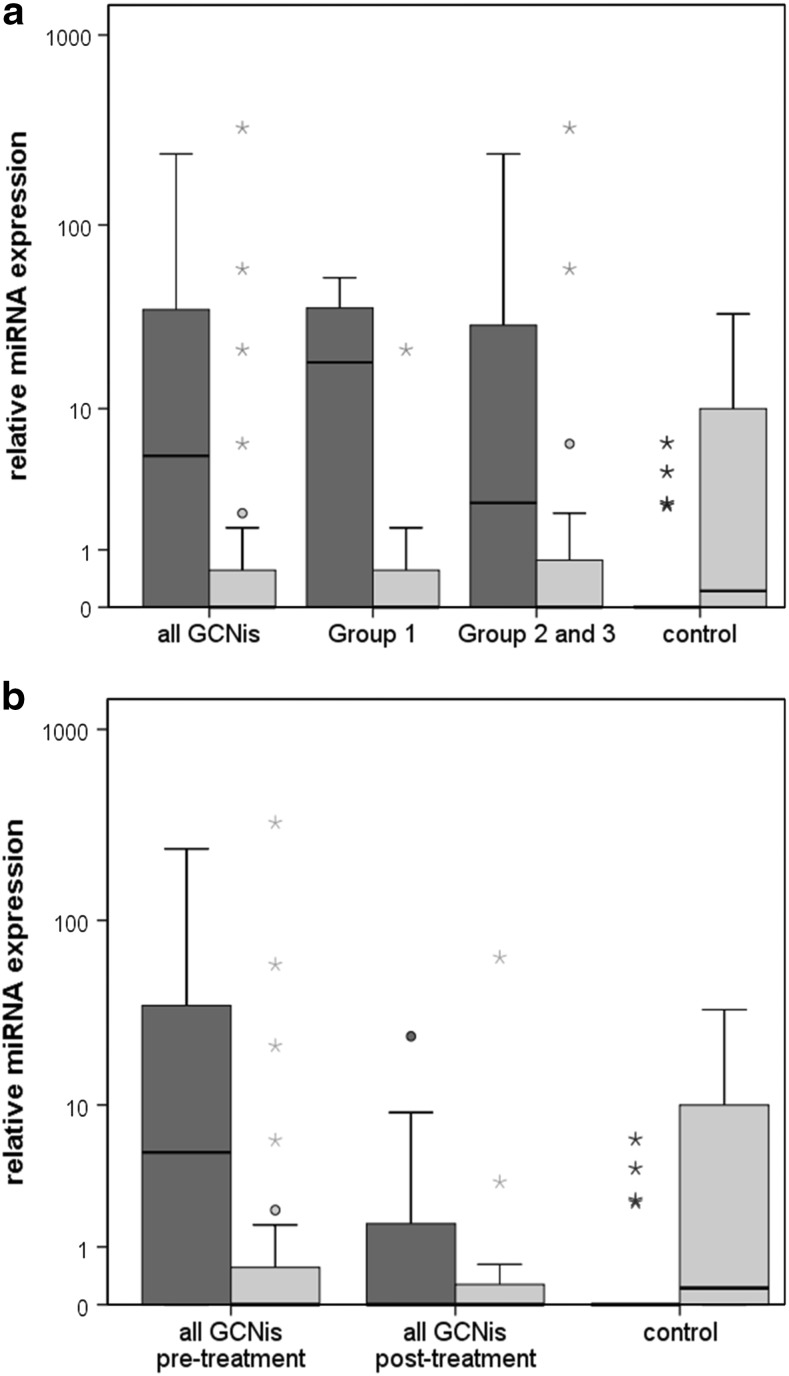
*Boxplots* of the relative expression of miR-371a-3p and miR-367-3p in GCNis patients and controls. **a** Relative miRNA expression in all patients (*n* = 27), patients of group 1 (*n* = 11) and group 2 and 3 (*n* = 16), and controls (*n* = 20). **b** *Boxplots* of the miR-371a-3p and miR-367-3p expression in patients before (*n* = 27) and after (*n* = 11) the treatment of GCNis. *Boxplots* of miR-371a-3p expression are depicted in *dark grey*, while *boxplots* of miR-367-3p expression are in *light grey*. The *y*-axis is plotted on a logarithmic scale

Figure 1a shows the median RQ values of the patient groups. In the entire GCNis group (all patients), the median miR-371a-3p expression was 5.2 (interquartile range, IQR = 35.8). The median serum level of miR-367-3p was 0.0 (IQR = 0.9) in the entire cohort.

The mean expression levels of miR-371a-3p and miR-367-3p of the GCNis subgroups are shown in Table 4. Noteworthily, one of the two patients with GCNis in both testes (group 3) had the highest miR-371a-3p expression of all patients with RQ = 237.18. The second patient with bilateral GCNis also had an expression (RQ = 35.8) above the 3rd quartile of group 2 and 3. Each of the GCNis group had significantly higher median expressions of miR-371a-3p than controls (Table 4), whereas median miR-367-3p expressions of the GCNis groups were not significantly different from controls.

**Table 4:** Relative expression of miR-371a-3p and miR-367-3p in GCNis subgroups

Subgroup	MiR-371a-3p			MiR-367-3p		
	Median RQ	IQR	Comparison with controls (*p* value)	Median RQ	IQR	Comparison with controls (*p* value)
All GCNis	5.2	35.8	0.001	0.0	0.9	0.167
Group 1	18.2	37.3	0.002	0.0	0.9	0.293
Groups 2 and 3	2.7	32.7	0.004	0.0	1.6	0.240
Controls	0.0	0.0	–	0.2	11.4	–

*IQR* inter quartile range, *RQ* relative quantity

### Serum levels after treatment of GCNis

**Fig. 2:**
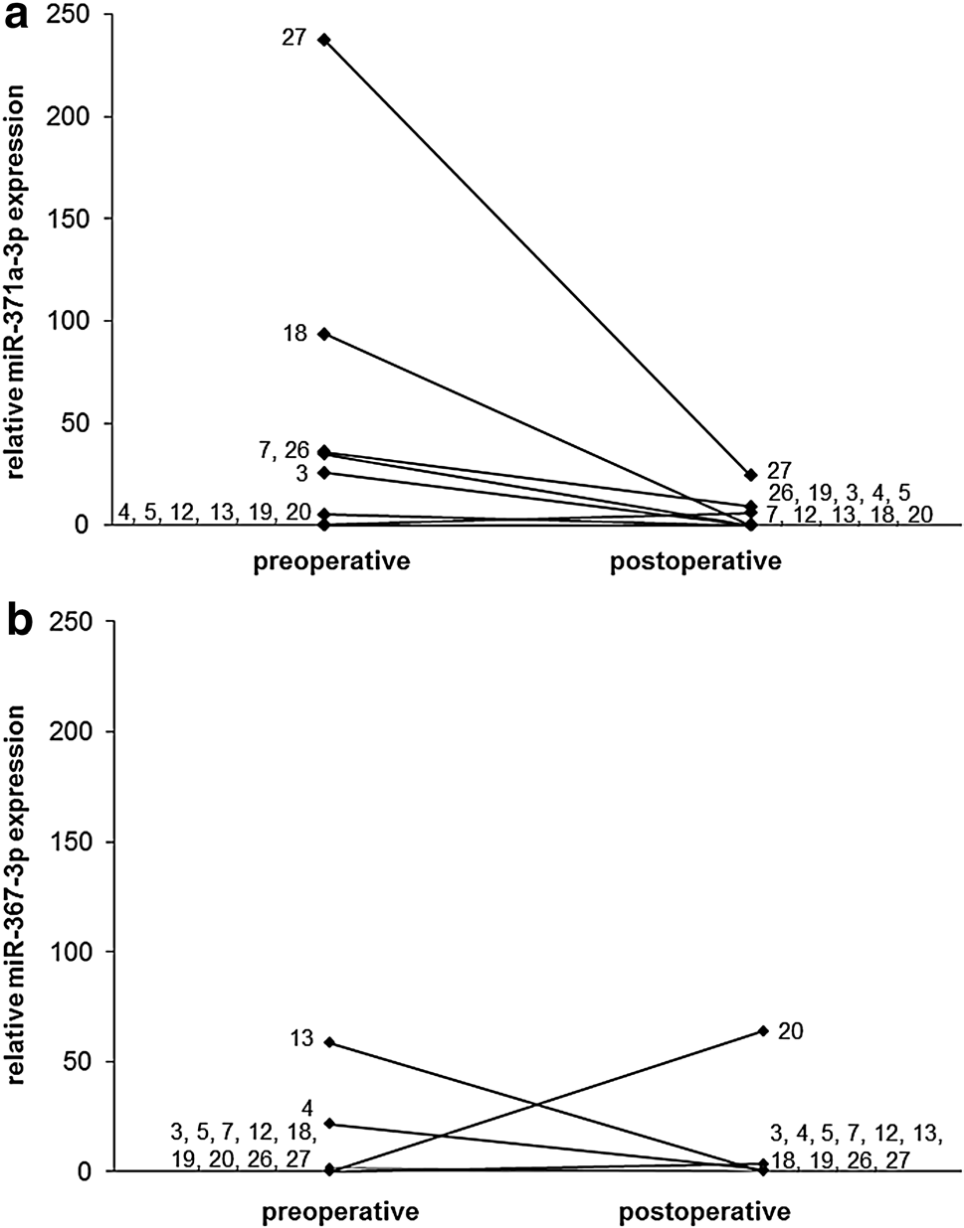
Individual results of the relative miRNA quantifications in GCNis patients. Comparison of data of expression in serum samples of 11 patients prior to surgery and postoperatively for miR-371a-3p (**a**) and miR-367-3p (**b**). The case no. indicated at the end of the lines is identical to Tables 1 and 2

Figures 1b and 2 show the results of repeat measurements after treatment of GCNis in 11 patients. After treatment, the median miR-371a-3p expression decreased to 0.0 (IQR = 6.0) which is significantly lower than the pre-therapeutic median value (*p* = 0.047). The median miR-367-3p expression was 0.0 (IQR = 0.6) after treatment which is not significantly different from the pre-therapeutic value. Figure 2 shows individual measurements of miR-371a-3p and miR-367-3p levels before and after treatment of GCNis. Apparently, all elevated serum levels of miR-371a-3p dropped to the normal range after treatment.

### Testicular vein serum levels

Levels of miR-371a-3p in testicular vein serum were manifold higher than in the corresponding cubital vein serum: patient 20: RQ = 57.3 vs. 0.0 and patient 23: 295.4 vs. 1.61 (Table 2). The testicular vein miR levels found in GCNis are considerably higher than the median level observed in testicular vein blood of healthy males (RQ = 4.3), as reported previously (Dieckmann et al. 2016).

### Results of in situ hybridisation

**Fig. 3:**
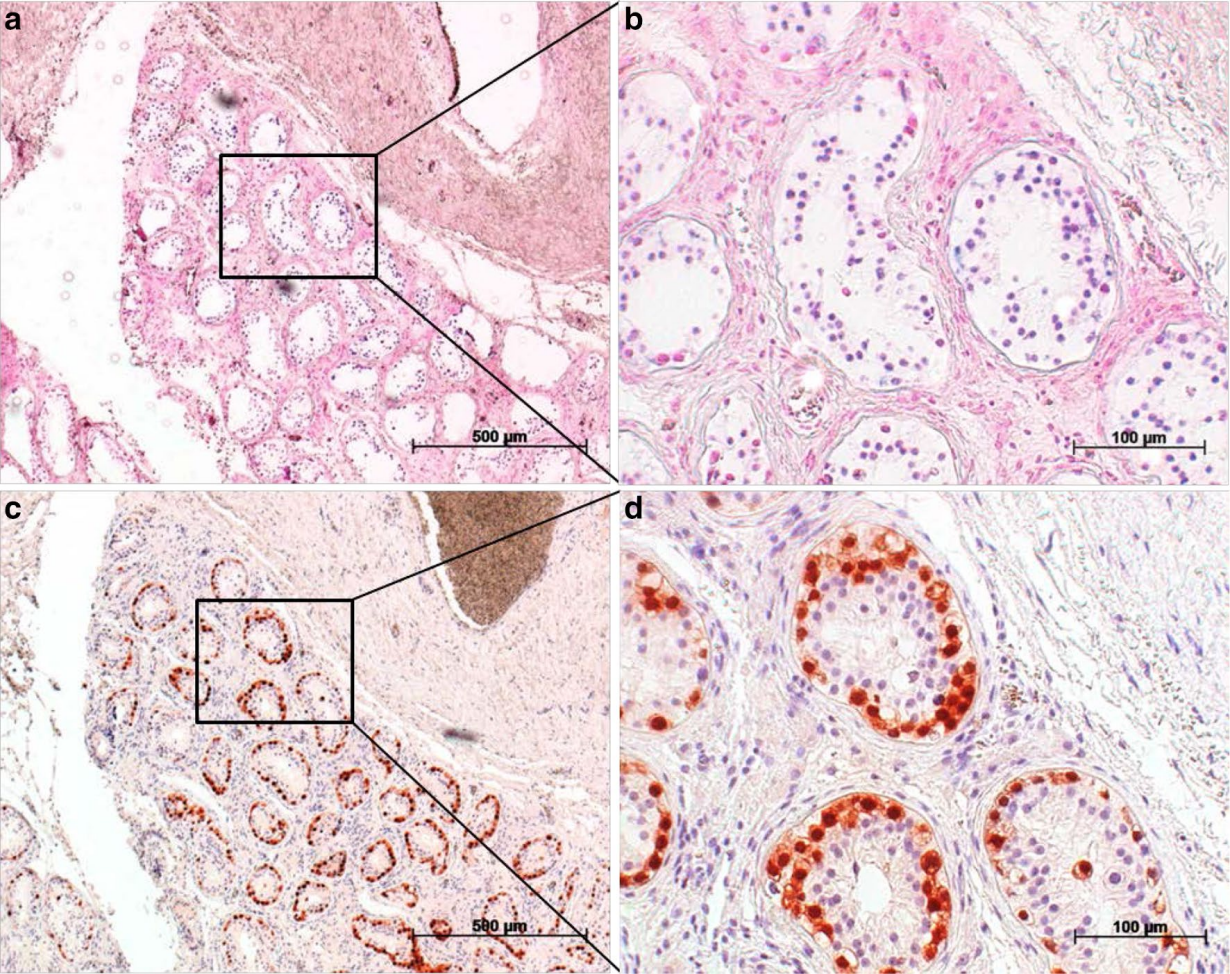
Detection of miR-371a-3p in GCNis via in situ hybridisation. **a** In situ hybridisation with a probe against miR-371a-3p causes blue staining in cells within the seminiferous tubules. **b** Section from (**a**). **c** Immunohistochemical staining of the same area with an Oct4 antibody. **d** Section from (**c**). Microphotographs **a** and **c** were taken at a ×50 magnification, microphotographs **b** and **d** were taken at ×200 magnification

Two of the four cases examined had morphological evidence of the presence of miR-371a-3p in GCNis cells. Figure 3 shows the result in case 1. The blue-stained intratubular cells represent GCNis expressing miR-371a-3p intracellularly (Fig. 3a, b). Identification of these cells as GCNis was achieved by additional staining with Oct4 (Fig. 3c, d). When identical areas of the sections revealed to be positive for both, Oct4 and the ISH probe, the presence of miR-371a-3p in GCNis was assumed.

## Discussion

There are two central results of the present study, first, the documentation of microRNA-371a-3p in GCNis cells, and second, the evidence of increased serum levels of miR-371a-3p in more than 50% of patients with GCNis.

The presence of miRNAs of the miR371-3 cluster in GCT tissue had been first demonstrated in 2006 (Voorhoeve et al. 2006; Looijenga et al. 2007) and several reports confirmed that finding, subsequently (Palmer et al. 2010; Gillis et al. 2007; Bing et al. 2012; Dieckmann et al. 2012). Regarding GCNis cells, Novotny et al. employed microarray techniques and FISH examination, and found the miR371-3 and 302/367 clusters to be significantly higher expressed in GCNis cells than in normal testicular tissue (Novotny et al. 2012). Our ISH analyses confirm the earlier reports. However, it is noteworthy that expression of miR-371a-3p in GCNis cells was found only in one half of our cases. Clearly, low sample size (*n* = 4) might have influenced this finding. On the other hand, the 50% intracellular detection rate correlates well with the 51.9% expression rate in serum as demonstrated in the present study (Table 2). Thus, despite the small sample size, it appears very probable that not all cases with GCNis will express the miR-371a-3p. Nonetheless, the documentation of intracellular miR-371a-3p molecules in GCNis cells supports the contention that the elevations of serum miR-371a-3p levels in the patients examined herein is specifically caused by GCNis cells and not by any unspecific reaction in the testicular parenchyma. In addition, the marked elevation of miR-371a-3p levels in testicular vein serum lends further evidence to the understanding that the circulating miR-371a-3p molecules originate from the testicular compartment.

We found elevated cubital vein serum levels of miR-371a-3p in roughly 52% of the patients. In most of whom, the elevation was only modest and much lower than the elevations caused by invasive GCTs (Syring et al. 2015; Pelloni et al. 2016). Our data are conflicting with results recently reported by a Dutch group and with preliminary data reported previously by our own group (van Agthoven and Looijenga 2016; Spiekermann et al. 2015). In both studies, a significant elevation of miR-371a-3p could not be detected in cubital vein serum of GCNis patients compared to controls. This discrepancy to our results is likely caused by the very low patient number in both studies (each *n* = 6).

Noteworthily, there are differences of median miR-371a-3p elevations between the three GCNis subgroups, however, these differences are not significant, statistically. At present, there is no biological pathway attainable to explain the differences. A chance effect resulting from small sample size could be considered.

The primary goal of the present study was to evaluate the diagnostic value of serum levels of miR-371a-3p for detecting GCNis in a non-surgical way. Clearly, the expression rate of 50% is too low to make serum levels of miR-371a-3p a promising tool for diagnosing GCNis without biopsy. Nonetheless, measuring the miR in serum could be an aid for guiding various clinical decisions. As miR-371a-3p in serum is highly specific for GCT and for GCNis, too, elevations of this miR could indicate the usefulness of a testicular biopsy in selected cases with particular risk factors, e.g. undescended testes, testicular microlithiasis, testicular atrophy, or familial testis cancer. Furthermore, in infertility clinics, the test could be used to guide decisions for or against testicular biopsy and also for the timing of radiation therapy particularly in cases on active surveillance attempting natural conception.

Furthermore, the test could probably be valuable for treatment control. As shown in the present study, all elevations of miR-371a-3p caused by GCNis dropped to the normal range after eradication. Thus, a post-therapeutic serum level below RQ = 5 would indicate the disappearance of GCNis obviating the need for a control biopsy.

It is particularly noteworthy that the two patients with bilateral GCNis had high miR-371a-3p serum levels. This result suggests a dose effect in the way that low numbers of GCNis cells will produce only little amounts of miR-371a-3p whereas higher numbers of GCNis cells (e.g. bilateral GCNis) will produce higher amounts of the miR, accordingly. It is known from quantitative histological studies that the number of GCNis cells may widely vary among patients afflicted with the lesion (Loy et al. 1990; van Casteren et al. 2008a, b). In some patients, GCNis cells may be distributed over the entire testis, whereas in other cases only few tubules of the testis are involved (Dieckmann et al. 2007). Thus, our finding of serum expression in only half of the GCNis cases would mirror the histological experience with the greatly variable distribution of the GCT precursor. An advantage of the serum test over testicular biopsy would be its independence of the individual morphological distribution of GCNis within the testicle.

One other hypothesis to explain the comparatively low detection rate of 50% with the serum test would relate to the inconsistent clinical behaviour of GCNis. This lesion is a quite inhomogeneous clinical entity and its biology is still incompletely understood. If GCNis is detected surgically, only 50% of the cases will progress to frank malignancy within 5 years (Skakkebaek et al. 1987). Some more cases will progress in the later course, but it is unknown to date if all of the cases with GCNis will definitely develop invasive GCT. Most probably, some do not. According to this well-established experience, one could speculate that GCNis comprises two different fractions of cells, one resting in a dormant stage with no ability to progression and the other fraction possessing the biological potential for transition to invasive GCT. While the biopsy will morphologically uncover all of the GCNis cells, the serum test could possibly reveal only those GCNis cases involving the potential for progression. According to this hypothesis, expression of miR-371a-3p in GCNis would highlight the gaining of one essential co-factor on the pathway to invasive GCT. Although this view would closely fit with our findings, it is clearly no more than a hypothesis and it definitely requires evidence by further studies.

One limitation to our study is clearly the small number of cases examined. However, patients with GCNis only in the testicle are quite rare. Merely 5–6% of GCT patients have contralateral GCNis (Kliesch et al. 2003; Dieckmann et al. 2007) and no more than 1% of infertile males (Rorth et al. 2000) are afflicted with the lesion. Therefore, the number of 27 patients involved in this investigation represents an appropriate sample size. Another possible limitation is that the co-existence of undetected small germ cell neoplasms in our GCNis cases cannot completely be ruled out in the cases not undergoing orchiectomy. Such minute co-existing tumours can easily escape clinical detection by sonography and palpation (Dieckmann et al. 2013), but would considerably increase the miR-371a-3p levels in serum. However, all cases had undergone thorough scrotal imaging examinations before including into this study, so the frequency of undetected small GCTs in the GCNis-afflicted testicles is certainly very low.

## Conclusions

The presence of miR-371a-3p molecules in GCNis cells was shown in two of four cases examined with in situ hybridization. Accordingly, elevated peripheral serum miR-371a-3p levels were documented in about one half of the patients with GCNis of the testis. Testicular vein serum levels of miR-371a-3p were much higher than in healthy males in both of the two cases examined. The expression rate of about 50% is too low to qualify measuring miR-371a-3p serum levels as a valuable method for primary diagnostics of GCNis. However, the method may aid in guiding the need for testicular biopsies in cases suspected to have the lesion. Possible candidates could be patients with testicular microlithiasis, adult males with undescended testis or infertile men. Moreover, the return of serum levels of miR-371a-3p to the normal range after treatment can indicate clearance of GCNis thus obviating the need of control biopsy. Presently, surgical testicular biopsy remains the standard way of searching for GCNis.

## Abbreviations

AFP: Alpha fetoprotein
bHCG: Beta human chorionic gonadotropin
FFPE: Formalin-fixed paraffin-embedded
GCNis: Germ cell neoplasia in situ
GCT: Germ cell tumour
IQR: Inter quartile range
ISH: In situ hybridisation
LDH: Lactate dehydrogenase
miR: micro RNA
RQ: Relative quantity
